# Hooked on fat: the role of lipid synthesis in cancer metabolism and tumour development

**DOI:** 10.1242/dmm.011338

**Published:** 2013-11

**Authors:** Franziska Baenke, Barrie Peck, Heike Miess, Almut Schulze

**Affiliations:** 1 Gene Expression Analysis Laboratory, Cancer Research UK London Research Institute, 44 Lincoln’s Inn Fields, London, WC2A 3LY, UK

## Abstract

An increased rate of lipid synthesis in cancerous tissues has long been recognised as an important aspect of the rewired metabolism of transformed cells. However, the contribution of lipids to cellular transformation, tumour development and tumour progression, as well as their potential role in facilitating the spread of cancerous cells to secondary sites, are not yet fully understood. In this article, we review the recent findings that support the importance of lipid synthesis and metabolism in tumorigenesis. Specifically, we explore the role of aberrant lipid biosynthesis in cancer cell migration and invasion, and in the induction of tumour angiogenesis. These processes are crucial for the dissemination of tumour cells and formation of metastases, which constitute the main cause of cancer mortality.

## Introduction

Cancer cells frequently exhibit specific alterations in their metabolic activity. This metabolic reprogramming supports the increased production of metabolic intermediates for the synthesis of proteins, nucleic acids and lipids, and is a prerequisite for the rapid proliferation of cancer cells. The most prominent metabolic alterations in cancer are an increase in glucose uptake and the use of aerobic glycolysis, termed the Warburg effect. However, other metabolic processes, including protein, nucleic acid and lipid biosynthesis, are also enhanced as part of cancer-associated metabolic reprogramming.

The majority of adult mammalian tissues satisfy their lipid requirements through the uptake of free fatty acids (FFAs) and lipoproteins, such as low-density lipoprotein (LDL), from the bloodstream. Fatty acid (FA) and cholesterol biosynthesis are restricted to a subset of tissues, including liver, adipose and lactating breast tissues. However, reactivation of lipid biosynthesis is frequently observed in cancer tissue (reviewed by Menendez and Lupu, 2007). Over the past few years, increasing attention has been given to the study of the metabolic processes involved in lipid biosynthesis and their regulation within the context of this disease. In this Review, we will highlight some of the recent evidence implicating deregulated lipid biosynthesis in cancer development. We will focus on the contribution of some of the key enzymes involved in FA and cholesterol biosynthesis to cell transformation and cancer development. We will discuss their regulation by oncogenic signalling pathways and by the environmental conditions in growing tumours. Finally, we will examine how deregulated lipid metabolism in cancer cells might contribute to the complex interactions between cancer cells and the variety of stromal cell types that are recruited into the tumour. Because stromal cells play a vital role in cancer development and disease progression, targeting lipid metabolism in cancer cells could have therapeutic benefits.

## Lipid synthesis in mammalian cells

**Fig. 1.**
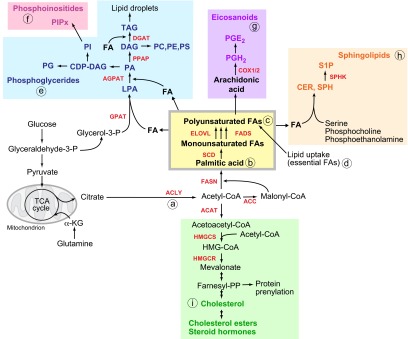
**Lipid biosynthesis.** Schematic overview of the pathways involved in the synthesis of fatty acids (FAs), cholesterol, phosphoglycerides, eicosanoids and sphingolipids. The enzymes involved in catalysing steps in lipid biosynthetic pathways are indicated in red. (a) Glucose- or glutamine-derived citrate is first converted to acetyl-CoA by ACLY. (b) For FA biosynthesis, acetyl-CoA is converted into malonyl-CoA. The repeated condensation of acetyl-CoA and malonyl-CoA by the multifunctional enzyme FASN leads to the generation of palmitic acid, a fully saturated 16-carbon FA. The introduction of a double bond in the Δ9 position of the acyl chain by SCD generates mono-unsaturated FAs. (c) Subsequent elongation and further desaturation produces the repertoire of FAs with different saturation levels. (d) Essential FAs (ω3 and ω6 FAs) cannot be synthesised by human cells and need to be provided from dietary sources. (e,f) Saturated and unsaturated FAs are combined with glycerol-3-phosphate (glycerol-3-P) to generate (e) phosphoglycerides and (f) phosphoinositides. (g) Arachidonic acid, a long-chain polyunsaturated FA, is used for the synthesis of eicosanoids. (h) Sphingolipids contain acyl chains and polar head groups derived from serine, phosphocholine or phosphoethanolamine. (i) Cholesterol biosynthesis is initiated by the conversion of acetyl-CoA to acetoacetyl-CoA. Addition of another acyl group by HMGCS produces 3-methylglutaryl-3-hydroxy-CoA, which is converted to mevalonate by HMGCR. Subsequent reactions result in the production of farnesyl-pyrophosphate, an essential intermediate for protein prenylation. Cholesterol also forms the structural backbone for steroid hormone biosynthesis. Enzyme abbreviations: ACAT, acetyl-CoA acetyltransferase; ACC, acetyl-CoA carboxylase; ACLY, ATP citrate lyase; AGPAT, 1-acylglycerol-3-phosphate O-acyltransferase; COX1/2, prostaglandin-endoperoxide synthase (PTGS); DGAT, diacylglycerol O-acyltransferase; ELOVL, fatty acid elongase; FADS, fatty acid desaturase; FASN, fatty acid synthase; GPAT, glycerol-3-phosphate acyltransferase; HMGCR, 3-hydroxy-3-methylglutaryl-CoA reductase; HMGCS, 3-hydroxy-3-methylglutaryl-CoA synthase; PPAP, phosphatidic acid phosphatase; SCD, stearoyl-CoA desaturase; SPHK, sphingosine-1-kinase. Metabolite abbreviations: α-KG, α-ketoglutarate; CDP-DAG, cytidine diphosphate-diacylglycerol; CER, ceramide; DAG, diacylglycerol; FA, fatty acid; LPA, lysophosphatidic acid; PA, phosphatidic acid; PC, phosphatidylcholine; PE, phosphatidylethanolamine; PG, phosphatidylglycerol; PGE_2_, prostaglandin E_2_; PGH_2_, prostaglandin H_2_; PI, phosphatidylinositol; PIPx, phosphatidylinositol phosphate; PS, phosphatidylserine; S1P, sphingosine-1-phosphate; SPH, sphingosine; TAG, triacylglyceride.

Lipid synthesis describes the processes that convert nutrient-derived carbons into FAs. The first step involved in FA and cholesterol biosynthesis is the production of two-carbon units in the form of acetyl-CoA. Acetyl-CoA is generated from citrate by the enzyme ATP-citrate lyase (ACLY) and then converted to malonyl-CoA by the enzyme acetyl-CoA carboxylase (ACC) (Fig. 1). Acetyl-CoA and malonyl-CoA are then coupled to the acyl-carrier protein domain of the multifunctional enzyme fatty acid synthase (FASN). Repeated condensations of acetyl groups generate a basic 16-carbon saturated FA: palmitic acid. Palmitic acid is further elongated and desaturated to generate the diverse spectrum of saturated and unsaturated FAs synthesised by mammalian cells. One of the main desaturases in mammalian cells is stearoyl-CoA desaturase (SCD), which introduces a double bond at the Δ9 position of palmitic and stearic acid to generate monounsaturated FAs. However, it should be noted that humans are not able to generate FAs that are unsaturated in the ω-3 or ω-6 position of the acyl chain. These essential FAs, α-linolenic acid and linoleic acid, need to be obtained from the diet.

FAs can be used to generate many different types of lipids. They are converted into diacylglycerides (DAGs) and triacylglycerides (TAGs) via the glycerol phosphate pathway, which uses the glycolytic intermediate glycerol-3-phosphate to form the glycerol backbone of these lipids. TAGs are mainly used for energy storage in the form of lipid droplets. However, intermediates of this pathway can be converted into different phosphoglycerides, including phosphatidylcholine (PC), phosphatidylethanolamine (PE), phosphatidylglycerol (PG) and phosphatidylserine (PS), which build the major structural components of biological membranes (Fig. 1).

Another class of lipids that are important for membrane function are sterols, mainly cholesterol and cholesteryl-esters (CEs). Cholesterol is synthesised from acetyl-CoA via the mevalonate pathway. Acetyl-CoA is first converted into acetoacetyl-CoA and then into mevalonate by the enzymes HMG-CoA synthase and reductase (HMGCS and HMGCR, respectively). The subsequent conversion of mevalonate to cholesterol involves the coordinated action of numerous enzymes that are part of this pathway. Cholesterol is an important membrane component because it modulates the fluidity of the lipid bilayer. In addition, it provides the structural backbone for the synthesis of steroid hormones such as estrogen and progesterone.

Other lipids that are generated from FAs are the sphingolipids, phosphoinositides and eicosanoids. Sphingolipids contain acyl chains coupled to serine and polar head groups derived from ethanolamine, serine or choline (Hannun and Obeid, 2008). Eicosanoids are produced from arachidonic acid, a polyunsaturated 20-carbon FA. Arachidonic acid is converted into prostaglandin H_2_ (PGH_2_) by prostaglandin H_2_ synthases, also known as cyclooxygenases (COX1 and COX2), or into leukotrienes by leukotriene synthases. PGH_2_ can be converted into additional prostaglandins, including prostaglandin E2 (PGE_2_), prostacyclin and thromboxanes (Wymann and Schneiter, 2008). Phosphoinositides, which contain two acyl chains coupled to an inositide head group, are important second-messenger molecules (Vanhaesebroeck et al., 2001; Wymann and Schneiter, 2008). Furthermore, sphingolipids, such as ceramide or sphingosine-1-phosphate, eicosanoids (e.g. PGE_2_) and phosphoinositides have important signalling functions in cells and tissues (Morad and Cabot, 2013; Wymann and Schneiter, 2008).

Most of the acetyl-CoA used for *de novo* FA and cholesterol biosynthesis is generated from glucose via the conversion of pyruvate to citrate in the tricarboxylic acid (TCA) cycle. However, recent evidence suggests that cancer cells are also able to generate citrate for FA biosynthesis through the reductive metabolism of glutamine (Metallo et al., 2012; Mullen et al., 2012; Wise et al., 2011). Moreover, direct incorporation of acetate could also contribute to the pool of cytoplasmic acetyl-CoA that is used for lipid biosynthesis in cancer cells, at least under certain conditions (Zaidi et al., 2012).

## The SREBP family: master regulators of lipid biogenesis

Because many cellular functions are dependent on the availability of lipids, lipid synthesis has to be regulated in a coordinated fashion to prevent lipotoxicity and membrane dysfunction (Poitout and Robertson, 2008; Santos and Schulze, 2012). Many genes coding for enzymes involved in FA and cholesterol biogenesis are targets of the sterol regulatory element-binding proteins (SREBPs), a family of transcription factors that are crucial for maintaining cellular lipid homeostasis (Bengoechea-Alonso and Ericsson, 2007). Aberrant activation of SREBPs can contribute to obesity, fatty liver disease and insulin resistance, and could also be involved in cancer development (Shao and Espenshade, 2012). SREBPs are produced as inactive precursors that reside in the membrane of the endoplasmic reticulum (ER), where they are associated with SREBP-cleavage activating protein (SCAP). Under limiting concentrations of sterols, the SREBPSCAP complex translocates to the Golgi, where SREBP is cleaved by two membrane-bound proteases, site-1 and site-2 protease (MBTPS1 and MBTPS2, respectively). This releases the N-terminal half of the SREBP protein, which contains the DNA-binding and transcriptional activation domains (Nohturfft and Zhang, 2008). SREBP1 also responds to reduced levels of the membrane phospholipid PC, which promotes the release of MBTPS1 and MBTPS2 to the ER membrane, thereby triggering proteolytic cleavage and nuclear translocation of SREBP1 (Walker et al., 2011).

Interestingly, SREBPs are also regulated downstream of growth factor signalling or in response to nutrient levels and the cellular energy status. It has been shown that the expression of many genes involved in cholesterol and FA biosynthesis is activated by Akt kinase, which mediates the effects of growth factor signalling via the phosphatidylinositol-3-kinase (PI3K) pathway (Porstmann et al., 2005). This regulation depends on the activity of mammalian target of rapamycin complex 1 (mTORC1), a multi-protein kinase complex that is regulated downstream of Akt (Düvel et al., 2010; Porstmann et al., 2008). SREBP is also a target of the AMP-regulated protein kinase (AMPK), a sensor of cellular energy levels (Hardie, 2007). AMPK phosphorylates SREBP and inhibits its proteolytic cleavage, thereby preventing hepatic steatosis and hyperlipidaemia in insulin-resistant mice (Li et al., 2011).

In addition to their regulated expression, the activity of several enzymes within the FA and cholesterol biosynthesis pathway is also modulated by growth factor signalling or in response to the cellular energy status. For example, ACLY is phosphorylated and activated by Akt (Berwick et al., 2002). Conversely, ACC and HMGCR are targets of AMPK, which phosphorylates and inhibits these enzymes in response to low cellular energy levels (Carling et al., 1989) (reviewed by Hardie, 2007).

## Aberrant lipid synthesis in cancer

An early study performed in the middle of the last century demonstrated that cancer tissues are able to generate lipids, including FAs and phospholipids, by *de novo* lipogenesis (Medes et al., 1953). These experiments found that tumour tissue shows levels of lipid biosynthesis that are comparable to that of liver tissue, which has a high rate of FA biosynthesis. Although Medes and colleagues noted that tumour tissue also takes up lipids from the tissue environment, they concluded that *de novo* lipogenesis provides the majority of lipids required for the rapid proliferation of cancer cells (Medes et al., 1953). Further evidence for lipid biosynthesis in cancer cells was provided by the observation that a tumour-specific antigen, OA-519, is indeed FASN (Kuhajda et al., 1994). Since this pivotal observation, numerous studies have confirmed that neoplastic tissues show aberrant activation of *de novo* lipogenesis and that inhibition of different enzymes within the FA biosynthesis pathway can block cancer cell growth (reviewed by Abramson, 2011; Igal, 2010; Menendez and Lupu, 2007; Santos and Schulze, 2012; Swinnen et al., 2006; Zaidi et al., 2012). Interestingly, an integrated analysis of gene expression data from breast cancer samples combined with a genome-scale human metabolic model indicated that FA biosynthesis is a feature of early stages of cancer development. More-advanced tumours show reduced proliferation but activation of an antioxidant signature (Jerby et al., 2012). This suggests that anabolic processes are more important during the initial expansion phase of early tumours whereas the advanced disease is more dependent on processes required for the detoxification of reactive oxygen species (ROS).

Most efforts to pharmacologically inhibit lipid synthesis have focussed on FASN, and the effects of inhibitors of this enzyme have been investigated in different preclinical cancer models (Kuhajda et al., 2000; Li et al., 2001; Puig et al., 2009; Zhan et al., 2008). FASN inhibitors have been shown to be effective in chemoprevention of breast cancer in *HER2/neu* transgenic mice (Alli et al., 2005) and in reducing the incidence of chemically induced lung tumorigenesis (Orita et al., 2008). Other enzymes within the FA biosynthesis pathway have also been targeted experimentally and were shown to limit the growth and proliferation of cancer cells; such enzymes include ACC (Beckers et al., 2007; Zhan et al., 2008) and SCD (Fritz et al., 2010; Mason et al., 2012; Roongta et al., 2011). Moreover, silencing of ACLY has been shown to block cancer cell growth both *in vivo* and *in vitro* (Bauer et al., 2005; Hatzivassiliou et al., 2005). SB-204990, an inhibitor of ACLY that has been shown to lower hepatic cholesterol and FA synthesis rates in rats (Pearce et al., 1998), also reduced tumour formation in lung and prostate xenografts (Hatzivassiliou et al., 2005; Migita et al., 2008). Interestingly, as well as being essential for FA synthesis, ACLY is also an essential regulator of histone acetylation, thereby linking cellular metabolism to the regulation of gene expression (Wellen et al., 2009). The relative contributions of these discrete roles of ACLY in tumorigenesis need to be more clearly defined to target ACLY effectively in anti-cancer therapies.

There is evidence that enhanced cholesterol biosynthesis plays a role in prostate cancer development (reviewed by Hager et al., 2006). Furthermore, dysregulation of the mevalonate pathway promotes cell transformation of primary mouse embryonic fibroblasts (Clendening et al., 2010). In addition, mutant forms of the p53 tumour suppressor can induce SREBP-dependent expression of enzymes in the cholesterol biosynthesis pathway (Freed-Pastor et al., 2012). By contrast, increased cholesterol biosynthesis and SREBP-dependent induction of N-Ras prenylation have been shown to contribute to the induction of DNA damage and senescence associated with the loss of the retinoblastoma tumour suppressor (*RB1*) in C-cell adenomas, thereby limiting progression to the malignant state (Shamma et al., 2009). Statins are potent inhibitors of HMGCR and are routinely used to lower cholesterol levels to prevent cardiovascular disease. Epidemiological studies have so far failed to show a clear effect of statin use on cancer incidence (Emberson et al., 2012), and additional clinical development is required to establish whether these drugs could provide an effective tool to target cholesterol biosynthesis in cancer.

Aberrant activation of SREBP and induction of expression of its target genes has been found in several cancer types, including breast, ovarian and prostate cancer (Swinnen et al., 2006). In prostate cancer, the expression of SREBPs and their targets changes during cancer progression (Ettinger et al., 2004). Interestingly, SREBP1 was found to regulate the transcription of the androgen receptor (*AR*) gene (Huang et al., 2010), and to promote proliferation, migration and invasion in prostate cancer (Huang et al., 2012). Furthermore, certain subtypes of glioblastoma multiforme (GBM) that express an activated mutant form of the epithelial growth factor receptor (EGFR) also display high levels of nuclear SREBP1. Treatment with inhibitors of FA and cholesterol biosynthesis reduced xenograft tumour formation in GBM cells engineered to express activated EGFR (Guo et al., 2009). Taken together, these findings strongly support a role of SREBP in tumour formation and cancer development.

## How does lipid synthesis promote cancer?

Despite the growing evidence demonstrating deregulated FA and cholesterol biosynthesis as features of cancer, the exact role of these metabolic alterations in the development and maintenance of the disease is not fully understood. Increased cancer cell proliferation requires the rapid synthesis of lipids for the generation of biological membranes. It is therefore not surprising that lipid biosynthesis is induced as part of the anabolic metabolism of cancer cells. Moreover, accumulation of energy-rich lipids could provide cancer cells with energy during times of nutrient depletion. However, there is also compelling evidence that the activation of lipid biogenesis could play a more active role in cell transformation and cancer development. We address these different possibilities in detail below.

## The structural roles of lipids

Lipids have important structural functions that are crucial for different aspects of the transformed phenotype. For example, cholesterol and other membrane lipids are required to form cholesterol-rich membrane rafts. These specialised structures are involved in membrane trafficking and act as essential platforms for the assembly of signalling complexes on the plasma membrane (Simons and Gerl, 2010). The biophysical properties of biological membranes are crucial for the functionality of membranous organelles, including the mitochondria and ER. Alterations in FA saturation can dramatically alter these properties and affect many aspects of the cellular machinery. It has been reported that the shift from lipid uptake to *de novo* lipogenesis in cancer cells leads to increased membrane lipid saturation, resulting in higher levels of saturated and monounsaturated phospholipids, potentially protecting cancer cells from oxidative damage by reducing lipid peroxidation (Rysman et al., 2010). Increased levels of saturated FAs, associated with reduced membrane fluidity, are also found in aggressive breast cancers, suggesting that reduced membrane fluidity is a feature of the advanced disease (Hilvo et al., 2011). However, inhibition of FA desaturation following ablation of SCD causes ER stress, cell cycle inhibition and apoptosis in cancer cells (Ariyama et al., 2010; Hess et al., 2010; Minville-Walz et al., 2010). Inhibition of SCD can also impair cancer cell proliferation by activating AMPK (Scaglia et al., 2009). Furthermore, depletion of SREBP1 and SREBP2 diminishes levels of monounsaturated FAs, resulting in mitochondrial dysfunction, the accumulation of ROS and ER stress in immortalised human epithelial cells (Griffiths et al., 2013). Interestingly, depletion of SREBP1 alone was sufficient to cause ER stress in GBM cells and blocked xenograft growth *in vivo* (Griffiths et al., 2013). These findings underline the importance of precisely controlled regulation of lipid synthesis and desaturation in cancer cells. Therapeutic strategies designed to inhibit lipid synthesis in cancer have to be carefully targeted to achieve the desired effect without harmful consequences for normal metabolic functions.

## Lipids as signalling molecules in cancer

In addition to their structural functions as membrane components, lipids are also important signalling molecules. Here, we focus on the signalling functions of lipid-derived molecules specifically in the context of cancer, and refer readers to earlier reviews for a more detailed overview of lipid signalling in normal and disease states (Hannun and Obeid, 2008; Wymann and Schneiter, 2008).

Phosphoinositides are important second messengers that relay signals from activated growth factor receptors to the cellular machinery. These molecules act as highly specific binding platforms for the recruitment of effector proteins to specific membrane compartments. One of the most prominent lipids of this class is phosphatidylinositol (3,4,5)-trisphosphate [PtdIns(3,4,5)*P*_3_; PIP_3_]. This molecule is produced by PI3K in response to growth factor signalling and mediates the recruitment and activation of Akt. PIP_3_ is also the substrate for phosphatase and tensin homologue (PTEN), and *PTEN* is one of the genes that is most frequently mutated or deleted in cancer (Li et al., 1997; Steck et al., 1997). Other lipid second messengers include lysophosphatidic acid (LPA), phosphatidic acid (PA) and DAG, which are produced by the action of different phospholipases. LPA, which can also be produced by the extracellular lysophospholipase autotaxin (Tokumura et al., 2002), activates cell proliferation, migration and survival through binding to G-protein-coupled receptors (Mills and Moolenaar, 2003).

Another important class of signalling lipids are the sphingolipids (Hannun and Obeid, 2008). Ceramide and sphingosine are produced in response to pro-apoptotic signals, including UV radiation or chemotherapy. Ceramide generally mediates growth inhibitory signals in cancer cells and is involved in the induction of apoptosis and growth arrest. Enzymes within the sphingolipid metabolism pathway are frequently deregulated in cancer, resulting in lower ceramide levels, which could cause resistance to chemotherapeutic treatment (see Morad and Cabot, 2013; and references therein). In contrast, sphingosine-1-phosphate (S1P) (produced from sphingosine by the action of sphingosine kinases) promotes cell proliferation, migration and angiogenesis (Morad and Cabot, 2013). PGE_2_ can activate intracellular signalling pathways, including the RAS-ERK pathway, and induce cancer cell proliferation in an autocrine fashion (Krysan et al., 2005). However, the main role of eicosanoids is likely to be the regulation of inflammation, which promotes tumour initiation and progression (Wang and Dubois, 2010). Aberrant activation of signalling pathways by oncogenes is likely to alter the abundance of multiple signalling lipids, thereby influencing numerous downstream processes that are crucial for cell transformation.

## Post-translational modification of proteins

Another important cellular function of lipids is in the post-translational modification of proteins. Acyl chains, most frequently palmitate or myristate, are covalently coupled to proteins on cysteine or N-terminal glycine residues (Resh, 2013). Moreover, prenyl groups, usually farnesyl or geranyl-geranyl groups, can be covalently attached to proteins to facilitate membrane association and protein-protein binding. Prenylation is essential for the correct localisation and activity of many signalling proteins. Farnesyl pyrophosphate, the precursor for farnesyl or geranyl-geranyl groups, is an intermediate of the cholesterol biosynthesis pathway. Cholesterol itself can also be coupled to proteins and is required for the subcellular regulation of Hedgehog, an essential regulator of development (Kornberg, 2011). Moreover, glycosylphosphatidyl inositol (GPI) anchors target secreted proteins to the outer leaflet of the plasma membrane (Paulick and Bertozzi, 2008).

The different types of lipid anchors are important for protein trafficking and subcellular localisation (Levental et al., 2010). Protein modification with saturated acyl chains can promote their association with cholesterol-rich membrane rafts, whereas unsaturated FAs exclude proteins from these structures. Thus, lipid modification is likely to have substantial effects on the activity of signalling complexes that are associated with membrane rafts. Moreover, protein acylation could be involved in the regulation of growth factor and cytokine secretion. A recent study showed that lysine-linked palmitoylation of tumour necrosis factor α (TNFα), an important modulator of apoptosis and the immune response, is controlled by the NAD-dependent deacetylase SIRT6 (Jiang et al., 2013).

## Lipids and autophagy

Recent studies have highlighted that lipid metabolism is also connected to autophagy, a mechanism of self-degradation that is required for the removal of defective proteins and organelles, which is induced under conditions of nutrient limitation. Lipids are integral components of the autophagic process and can impact it at different levels (reviewed by Dall’Armi et al., 2013). For example, one of the initiating steps of autophagy involves the covalent binding of PE to the autophagy-related protein Atg8 (LC3). Interestingly, the release of lipids from lipid droplets in response to nutrient starvation requires components of the autophagic machinery, including Atg5, and inhibition of autophagy results in lipid droplet accumulation (Singh et al., 2009). This mode of mobilisation of lipids from intracellular stores has consequently been termed ‘macrolipophagy’ (Singh et al., 2009). Furthermore, a number of genes involved in autophagy are transcriptionally regulated by SREBP2, and it has been shown that SREBP2 is required for the induction of autophagy after lipid depletion (Seo et al., 2011). The link between lipid metabolism and autophagy is particularly interesting as increasing evidence confirms the importance of autophagy in cancer. It has been shown that autophagy allows Ras-transformed cells to maintain their energy supply during nutrient starvation and tumorigenesis (Guo et al., 2011). These results suggest that autophagy can function as a pro-survival pathway in cancer (White, 2012).

In light of the diverse roles of lipids in membrane structure, cellular signalling and protein regulation, it is obvious that lipids are essential components of the cellular machinery that regulates proliferation, migration and survival of cancer cells. Nonetheless, it is less clear whether simply enhancing the rate of FAs and cholesterol biosynthesis, as a consequence of the activation of SREBP, FASN or other lipid-synthesis enzymes, directly promotes tumorigenesis. Altered lipid synthesis and modification in cancer could affect the production of specific signalling lipids, such as those derived from poly-unsaturated FAs, or affect the availability of specific pools of FAs for protein modification. Differences in the length and saturation levels of acyl chains could also affect accessibility or subcellular localisation of phosphoinositides, thereby modulating the output of growth-promoting signalling pathways. Recent technological advances, such as the accurate detection and quantitation of numerous lipid species by mass spectrometry, allow the profiling of discrete changes within the lipid composition of cancer cells and will help to elucidate the intricate connections between lipid metabolism and cell function in cancer.

## Lipids and the tumour microenvironment

Epithelial cancer development and progression can be viewed as a multi-step process, during which the normal tissue architecture is disrupted by the expanding cancer cell population. Induction of cell migration and matrix degradation results in cancer cells breaking away from the confines of the epithelium to initiate the formation of a tumour. In addition, activated fibroblasts and bone-marrow-derived stromal cells are recruited to the tumour and promote its progression to the malignant state. Together with the environmental conditions created by the expanding cell mass, stromal cells constitute an important component of the tumorigenic microenvironment in which cancer cells exist (Hanahan and Coussens, 2012). The following sections will discuss the potential impact of deregulated lipogenesis in the context of the complex interactions between cancer cells and the tumour microenvironment.

## Disruption of normal tissue architecture

**Fig. 2.**
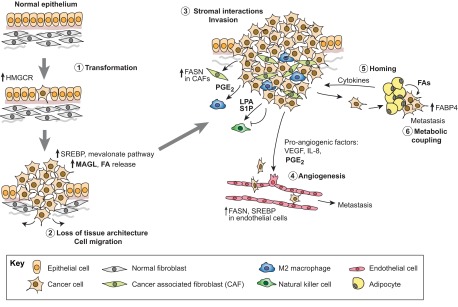
**The roles of lipids in the tumour microenvironment.** Lipids play important roles during tumour initiation and disease progression. Activation of HMGCR drives cell transformation (stage 1). Activation of SREBP and induction of enzymes of the mevalonate pathway are involved in the disruption of normal tissue architecture, and release of FAs by MAGL can promote cancer cell migration (stage 2). Lipids are also involved in the interaction of cancer cells with components of the tumour stroma. For example, cancer-associated fibroblasts (CAFs) show increased expression of FASN. Signalling lipids, including PGE_2_, regulate the recruitment of cancer-promoting M2 macrophages, which can promote cancer cell migration and invasion (stage 3). LPA and S1P regulate the cytotoxic function of natural killer (NK) cells. PGE_2_ can also act as a pro-angiogenic factor by promoting the outgrowth (sprouting) of vascular and lymphatic endothelial cells, and the dissemination of tumour cells into distant tissues during metastasis formation (stage 4). Adipocytes can induce the homing of metastatic cancer cells by releasing cytokines (stage 5). The metabolic coupling between adipocytes and cancer cells involves the release of FAs by adipocytes, which are then used for energy production by cancer cells (stage 6).

The breakdown of normal tissue architecture is a hallmark of cancer (Fig. 2, stages 1 and 2). Polarised epithelia are converted into disorganised structures that can invade the surrounding tissues. These early events in cancer development can be recapitulated *in vitro* by analysing the growth of cancer cells in three-dimensional (3D) cultures. For example, normal mammary epithelial cells form polarised structures, called acini, whereas most breast cancer cells grow in a non-polarised and invasive manner. This disruption of normal mammary tissue architecture can be mediated by cancer-associated mutant forms of p53 (Freed-Pastor et al., 2012). The same study that demonstrated that mutant p53 proteins activate the mevalonate pathway by binding to SREBP (described above), also showed that inhibition of different enzymes within this pathway blocks the invasive morphology of breast cancer cells grown in 3D, potentially by interfering with geranyl-geranylation of small GTPases (Freed-Pastor et al., 2012). Aberrant FA synthesis has also been linked to the disruption of cell polarity in epithelial tissues. Expression of active SREBP1c in *Xenopus* embryos results in aberrant tissue morphology and causes the loss of the primary cilium from epithelial cells (Willemarck et al., 2010). This specialised microtubular structure is expressed on the apical surface of normal epithelial cells and is lost in many cancers. Moreover, SREBP activation also disturbed cell polarity in Madin-Darby canine kidney cells and normal tissue development in the prostate (Willemarck et al., 2010). Together with the earlier observations that HMGCR can induce anchorage-independent growth in breast epithelial cells (Clendening et al., 2010), these findings clearly highlight the importance of the mevalonate pathway during the early stages of cancer development.

## Cancer cell migration

Directed cell migration requires the coordinated activation of several processes: cell polarisation and elongation, formation of cell protrusions and attachment to components of the ECM, and contraction of the cell body to generate a force for the movement of the cell body in the direction of the leading edge (Friedl and Wolf, 2003). Cell migration is induced in response to pro-migratory factors, including growth factors and chemokines but also by signalling lipids, such as DAG, LPA and prostaglandins (Fig. 2, stage 2). The role of these signalling lipids in cell migration is well documented and has been reviewed elsewhere (Park et al., 2012). However, it is less clear how global changes in lipid composition might modulate the migratory capacity of cancer cells. A recent study identified a specific lipid signature that was associated with overexpression of monoglyceride-lipase (MAGL), a lipase involved in releasing FFAs from TAGs (Nomura et al., 2010). Overexpression of MAGL was found in highly aggressive cancers and its inhibition caused defects in cancer cell migration and tumour growth. Interestingly, the reduction in tumour growth following MAGL inhibition was completely rescued when animals were fed a high-fat diet (Nomura et al., 2010). This finding suggests that diet-derived lipids can contribute to the global lipid composition of cancer cells and might affect complex signalling events within tumours. It also raises the question as to whether therapeutic strategies that target cancer lipid metabolism would only be effective in combination with stringent dietary regimes.

## Tumour-stromal interactions

Tumour growth is also strongly affected by the complex interactions between cancer cells and stromal components (Joyce and Pollard, 2009), including cancer-associated fibroblasts (CAFs), macrophages and other immune cells (Fig. 2, stage 3). Expression of FASN is induced following activation of G-protein-coupled estrogen receptor (GPER) and supports cell proliferation in CAFs (Santolla et al., 2012). Different types of macrophages can modulate cancer growth in different ways. Macrophages of the M1 type induce a pro-inflammatory programme that can inhibit tumour growth by inducing the recognition of tumour cells by the immune system. In contrast, M2 macrophages generally promote tumour growth by inducing cancer cell invasion, angiogenesis and metastasis formation (Joyce and Pollard, 2009). The interaction between tumour cells and stromal components is mediated by secreted factors, including growth factors and chemokines. Prostaglandins are important modulators of the immune response, and inhibition of PGE_2_ synthesis using COX2 inhibitors in cervical cancer cells was shown to prevent the differentiation of monocytes into M2 macrophages (Heusinkveld et al., 2011). In addition, induction of FA biosynthesis is a key requirement for the phagocytic function of macrophages (Ecker et al., 2010) and SREBP1 is involved in the regulation of macrophage function within the innate immune response (Im et al., 2011). LPA and S1P are also important factors in the interaction between cancer cells and the immune system because they can modulate the recruitment and cytotoxicity of natural killer (NK) cells (Rolin and Maghazachi, 2011). Lipids thus take a central role in the communication between cancer cells and stromal components during tumour development and progression.

## Induction of angiogenesis

Another important mechanism that is essential for tumour growth is the induction of angiogenesis (Fig. 2, stage 4). This is required to maintain a supply of nutrients and oxygen once the tumour has outgrown the diffusion limits of these factors. Tumour vasculature is also required for the removal of metabolic end products, such as lactic acid, that could have toxic effects on tumour cells. However, tumour vasculature is often characterised by an aberrant structural organisation of its vessels, which results in high vascular permeability. *De novo* hemangiogenesis and lymphangiogenesis also allow the dissemination of tumour cells into distant tissues, thereby facilitating the formation of metastases. Interestingly, both processes were shown to be stimulated by S1P in a murine model of metastatic breast cancer (Nagahashi et al., 2012). Furthermore, it has been shown that the secretion of PGE_2_ by breast cancer cells induces an angiogenic switch through both paracrine and autocrine mechanisms. PGE_2_ promotes angiogenesis by stimulating proliferation and tube formation in endothelial cells. In addition, it binds to G-protein-coupled receptors on tumour cells to induce the production of pro-angiogenic factors, including vascular endothelial growth factor (VEGF) and angiogenin (ANG) (Chang et al., 2004). Metastasis formation is also promoted by the production of prostaglandins in endothelial cells of the lymphatic system in response to lymphangiogenic growth factor signalling (Karnezis et al., 2012).

The importance of lipid metabolism in angiogenesis is further highlighted by the observation that endothelial cells activate SREBP1 and SREBP2 following exposure to VEGF (Zhou et al., 2004). Moreover, SREBP1 was found to be abundant in newly developed microvasculature (in a rabbit skin wound-healing model) (Yao et al., 2006). In addition, treatment with 25-hydroxycholesterol (25-HC), a potent inhibitor of SREBP processing, prevented VEGF-induced proliferation and migration in human microvascular endothelial cells (hMVECs) and blocked blood vessel formation in chick embryos (Zhou et al., 2004). Conversely, pro-angiogenic factors, such as FGF, IL-8, thrombin and TGFβ, induce SREBP activation and IL-8-induced angiogenesis is blocked by treatment with 25-HC (Yao et al., 2006). SREBP also seems to be involved in the induction of endothelial cell migration in response to insulin by mediating the activation of Rac downstream of Akt (Liu et al., 2009). Moreover, FA synthesis itself has also been linked to tumour angiogenesis, because treatment with the FASN inhibitor orlistat reduced the formation of lung metastases in a melanoma model (Seguin et al., 2012). Surprisingly, in this study, the production of VEGFA by cancer cells was actually increased in response to orlistat. However, inhibition of FASN reduced proliferation and viability of rat aortic endothelial cells and human umbilical vein endothelial cells (RAECs and HUVECs), and blocked their capacity to form capillary-like structures in culture (Seguin et al., 2012), suggesting that the inhibition of metastasis formation by orlistat is caused by its effects on endothelial cell metabolism. These findings are particularly interesting because endothelial cell metabolism is increasingly considered as an attractive target for cancer therapy (Harjes et al., 2012).

## Lipid metabolism and hypoxia

The studies mentioned above provide evidence for the importance of cholesterol and FA biosynthesis in endothelial cell metabolism and suggest that inhibitors of these pathways might be useful in the clinic. In light of the evidence linking FA synthesis to oncogene signalling, it is possible that activation of SREBP and FASN could also stimulate processes involved in endothelial cell recruitment and induction of tumour vasculature by the cancer cells. One tissue environmental factor that contributes to tumour angiogenesis is low oxygen (hypoxia). Once a tumour has outgrown the tissue blood supply, the reduced concentration of oxygen stimulates the stabilisation and activation of the hypoxia-inducible factor (HIF), a transcription factor that is the main mediator of the cellular response to hypoxia (Kaelin and Ratcliffe, 2008). Hypoxia-dependent activation of HIF induces a number of metabolic alterations, most notably the induction of glycolysis and the inhibition of mitochondrial oxidative phosphorylation (Denko, 2008). The hypoxia-dependent regulation of lipid metabolism is not fully understood. It has been shown that hypoxic cells use glutamine as a major substrate for lipid synthesis (Metallo et al., 2012; Wise et al., 2011). Activation of HIF2α, one of the three isoforms of the oxygen-sensitive α-subunit of HIF, blocks lipogenesis and FA β-oxidation in liver cells while promoting lipid storage, thereby contributing to fatty liver disease (Rankin et al., 2009). However, cancer cells seem to retain some level of β-oxidation under hypoxia, shown by the requirement for CPT1C, an isoform of carnitine palmitoyltransferase (which is required for the transport of long-chain FAs into mitochondria for degradation), to support cancer cell survival under hypoxic stress (Zaugg et al., 2011).

Interestingly, the SREBP pathway functions as an oxygen sensor in yeast (Hughes et al., 2005). Cholesterol biosynthesis, which requires molecular oxygen, is inhibited under hypoxic conditions and the resulting drop in cholesterol concentration causes enhanced proteolytic processing and activation of Sre1, the yeast homologue of mammalian SREBP (Hughes et al., 2005). Moreover, it has been reported that FASN expression is induced in response to hypoxia through the activation of Akt and SREBP1, and high levels of FASN expression were found in hypoxic regions of breast cancer xenografts (Furuta et al., 2008). In contrast, another report showed that FA biosynthesis is unaffected by hypoxia in prostate cancer cells (Higgins et al., 2009).

In addition to regulating FA biosynthesis, hypoxia can also influence the lipid composition of cancer cells by affecting pathways involved in lipid synthesis and modification as well as altering the uptake of lipids from the environment. It has been shown, for example, that HIF1α and the peroxisome proliferator-activated receptor γ (PPARγ), a transcription factor involved in the regulation of genes involved in lipid metabolism, mediate the induction of lipid uptake and modification through the glycerol phosphate pathway in cardiac hypertrophy (Krishnan et al., 2009). More recently, a study showed that hypoxic cancer cells take up lysophospholipids to fulfil their demand for unsaturated FAs (Kamphorst et al., 2013).

Lipid modification can also be affected by oxygen availability. Exposure to intermittent hypoxia leads to the induction of SREBP1, SCD1 and SCD2 in mice (Li et al., 2006). Furthermore, SCD1, the main isoform of SCD in humans, is induced by hypoxia in clear cell renal carcinoma cells and regulates the expression of HIF2α through a positive feedback loop involving Akt (Zhang et al., 2013). As already discussed above, changes in lipid desaturation mediated by altered SCD expression could affect membrane fluidity and contribute to the induction of cancer cell migration, a process that is also regulated by hypoxia. In addition, the activity of many signalling lipids involved in the crosstalk between cancer cells and the vasculature could also be affected by FA desaturation.

## Tumour cell dissemination and metabolic coupling

The generation of metastatic cancer does not only depend on the growth of blood or lymphatic vessels into the primary tumour, but also the migration of cancer cells to distant sites (Fig. 2, stage 5). Circulating cancer cells also need to extravasate and be able to proliferate in the respective tissue. The signals that determine the homing of cancer cells to different tissues are so-far poorly described. A recent study identified an interesting connection between lipid metabolism and metastasis formation. Ovarian cancer cells preferentially metastasise to the omentum, an abdominal fat pad. The study found that omental adipocytes promote ovarian cancer cell homing to the omentum through the induction of specific, adipocyte-derived cytokines (adipokines) (Nieman et al., 2011). Conversely, ovarian cancer cells activate lipolysis within the adipocytes and use adipocyte-derived lipids for β-oxidation, thereby obtaining energy for tumour growth (Fig. 2, stage 6). The coupling of energy metabolism between metastatic cancer cells and their surrounding adipocytes was found to be dependent on the function of fatty acid binding protein 4 (FABP4), expression of which is upregulated in omental metastases compared with the primary tumour (Nieman et al., 2011). Growth-promoting capacities of adipocytes have also been described for breast and prostate cancer (Klopp et al., 2012; Manabe et al., 2003; Tokuda et al., 2003). Because it is likely that other stromal cells also contribute to the energy metabolism of cancer cells, targeting the energetic coupling between cancer and stroma provides an additional therapeutic opportunity.

## Conclusions and implications for the clinic

Lipid metabolism has now been accepted as a major metabolic pathway that is involved in many aspects of cancer cell biology. In addition to the synthesis of DNA and proteins, production of lipids is a prerequisite for cell growth and proliferation. However, lipids are also active players in the signalling processes that are involved in cell transformation and tumour development. Deregulated lipid metabolism has been demonstrated in many cancer settings. In light of the large repertoire of different lipid classes and variation in length and saturation of the acyl chains, attempts to understand the role of different lipids in cancer physiology depend on the ability to accurately monitor alterations in cellular lipid composition. Detailed analysis of intact tumour tissue is required to determine the complete spectrum of lipids within cancer cells. Increasingly, lipidomics approaches are being applied to analyse the lipid composition of cancer cell lines or fresh tumour specimens by mass spectrometry (for example, see Hilvo et al., 2011). Positron emission tomography (PET) with acetate or choline tracers is used to visualise active lipid synthesis in tumours (Zhu et al., 2012) and could be used for the dynamic monitoring of response to treatments targeting lipid metabolism. In addition, because cancer cells might be able to obtain some rate-limiting lipids through uptake from the surrounding tissue, it is also essential to identify the lipid composition of the tumour microenvironment. One example is a study in which nuclear magnetic resonance (NMR) spectroscopy was used to analyse the levels of cholesterol, cholesterol esters and phospholipids in human GBM samples. A comparison of these lipid levels in tumour tissue, cerebrospinal fluid and serum revealed differences between tumour grades and could be useful as a diagnostic tool (Srivastava et al., 2010).

Moreover, the effect of blocking individual components of the pathways involved in the biosynthesis, uptake or remodelling of lipids needs to be evaluated not only in the context of cancer cell proliferation and survival but also within the more complex setting of cancer cell migration, invasion, tumour angiogenesis and metastasis formation. A number of chemical inhibitors of lipid biosynthesis, most prominently inhibitors of FASN, have been investigated in preclinical cancer models or are entering clinical trials. Some of these drugs have originally been developed to treat metabolic diseases, such as diabetes, hyperlipidaemia or the metabolic syndrome, and it is likely that more overlap between these disease settings and cancer will be discovered in the future. In this context, the contribution of dietary factors, such as saturated and unsaturated FAs, also needs to be considered – future cancer therapeutic strategies could incorporate strict dietary regimes. Despite the substantial challenges that need to be overcome for successful drug development, a plethora of novel therapeutic opportunities are indicated.
